# The Culture-Transmission Motive in Immigrants: A World-Wide Internet Survey

**DOI:** 10.1371/journal.pone.0141625

**Published:** 2015-11-03

**Authors:** Irina Mchitarjan, Rainer Reisenzein

**Affiliations:** 1 Institute of Educational Science, University of Greifswald, D-17487 Greifswald, Germany; 2 Institute of Psychology, University of Greifswald, D-17487 Greifswald, Germany; National Institute of Health, ITALY

## Abstract

A world-wide internet survey was conducted to test central assumptions of a recent theory of cultural transmission in minorities proposed by the authors. 844 1^st^ to 2^nd^ generation immigrants from a wide variety of countries recruited on a microjob platform completed a questionnaire designed to test eight hypotheses derived from the theory. Support was obtained for all hypotheses. In particular, evidence was obtained for the continued presence, in the immigrants, of the culture-transmission motive postulated by the theory: the desire to maintain the culture of origin and transmit it to the next generation. Support was also obtained for the hypothesized anchoring of the culture-transmission motive in more basic motives fulfilled by cultural groups, the relative intra- and intergenerational stability of the culture-transmission motive, and its motivating effects for action tendencies and desires that support cultural transmission under the difficult conditions of migration. Furthermore, the findings suggest that the assumption that people have a culture-transmission motive belongs to the folk psychology of sociocultural groups, and that immigrants regard the fulfillment of this desire as a moral right.

## Introduction

In the wake of increasing international migration, the question of how majority societies deal with linguistic and cultural, ethnic and religious minorities and the related issue of cultural transmission in minorities, have become important topics of research in several branches of social science including sociology, psychology, and educational science (e.g. [1–7]). Our research focuses on the pedagogical aspects of cultural transmission in minorities: the educational activities of minorities in a cultural majority environment, and the policies of the majority towards them. Past attempts to explain these social phenomena have for the most part tried to apply existing, broad theoretical frameworks to them. Mostly taken from sociology, these comprise in particular different versions of assimilation theory (e.g. [8–11]), transnationalism [12], and Bourdieu’s [13] theory of cultural capital. However, in part simply because these theories were not originally developed to explain cultural transmission in minorities, they provide, in our view, only partial explanations (for more detail, see [14,15]).

As an alternative, Mchitarjan and Reisenzein [14,16]; see also, [17,18]) proposed a theory of cultural transmission in minorities that is tailored to the social phenomena in question. This theory combines an action-theoretical model, adopted from sociology and social psychology, with a theory of the cultural evolution of groups, and fills in this theoretical framework with specific assumptions about the motives and strategies of majorities and minorities in cultural transmission situations. A central assumption of the theory is that members of sociocultural groups have a culture-transmission motive, that has emerged in cultural evolution and is activated in migration situations. This plus other assumptions, allow the theory to explain several migration-related phenomena that pose difficulties to existing accounts, in particular the frequently observed stability of cultural traits in immigrants and autochthonous ethnic minorities (e.g. [1,7,15]).

Several predictions of the theory of cultural transmission in minorities are at least indirectly supported by existing historical studies of migration processes (e.g. [19,20]) and by surveys of migrants (e.g. [21–23]). A first direct empirical test of the theory was conducted by Mchitarjan and Reisenzein [5] with a small sample of adolescents and young adults with mostly Russian migration background in Germany. In the present article, we report the results of an internet survey that tested the theory with a much larger sample of 1^st^-2^nd^ generation immigrants stemming from a wide variety of countries and cultures. Eight hypotheses contained in, or derived from the theory were tested (see Table 1). In the following section, we give a summary of the theory, focusing on those of its assumptions that were tested in our study.

**Table 1:** Hypotheses and Main Supportive Findings.

**Hypothesis 1.** Immigrants (like all sociocultural groups) have a culture-transmission motive, the desire to maintain and transmit their culture of origin. Specifically, this desire is still present in the majority of 1^st^ to 2^nd^ generation immigrants.
**Supportive findings.** Hypothesis 1 is supported by the high scores obtained on a direct measure of the culture-transmission motive (CTM-Desire), and more indirectly by similarly high scores obtained on two indirect motive indicators (CTM-Emotion and CTM-Action).
**Hypothesis 2.** The culture-transmission motive focuses on the values and norms of the culture of origin, as well as on reliable outward signs of cultural identity, in particular the language.
**Supportive findings.** Hypothesis 2 implies that items measuring wishes to transmit specific core elements of culture (language, values and norms) and items measuring the desire to transmit the culture in general, are strongly intercorrelated. Supporting this prediction, a factor analysis of the specific and general motive items included in the questionnaire revealed a strong common factor on which all items had from moderate to high loadings. Additional, indirect support for hypothesis 2 is provided by the substantial correlations of the culture-transmission motive to (a) the readiness of immigrants to take countermeasures against the threats of language loss and cultural estrangement of their child and (b) the desire to see the culture of origin considered in the public school system of the resident country (see hypothesis 5).
**Hypothesis 3.** The culture-transmission motive is based on, or derived from, more basic motives fulfilled by social groups.
**Supportive findings.** Supporting hypothesis 3, most participants ascribed to their culture of origin all of a set of frequently proposed social functions of groups to some degree (providing the individual with a sense of identity, cognitive guidance, security and protection, and the appreciation and support of the group members), and the desire to transmit the culture of origin could be well predicted from these perceived instrumentalities (multiple *R* = .72).
**Hypothesis 4.** The culture-transmission motive is (4a) intragenerationally highly stable and (4b) intergenerationally as stable as are core transmitted norms and values.
**Supportive findings.** The predicted intragenerational stability of the culture-transmission motive (operationalized as its resistance to change) was supported by its low correlation to the participants’ length of stay in the country of residence (*r* = -.10). The predicted intergenerational stability was supported by a correlation of *r* = .67 between the culture-transmission motive of the participants and that of their parents (as reported by the participants).
**Hypothesis 5:** The culture-transmission motive is activated in the migration situation and then (5a) motivates actions (specifically of a pedagogical nature) and (5b) generates desires that help immigrants to cope with the increased difficulty of cultural transmission under the conditions of migration.
**Supportive findings**. Supporting hypothesis 5a, the strength of the immigrants’ culture-transmission motive significantly predicted their readiness to take countermeasures against (a) the perceived cultural estrangement of their child and (b) the unavailability of instruction in the language of origin in the public schools. Supporting hypothesis 5b, the strength of the immigrants’ culture-transmission motive significantly predicted (c) their wish that the language, history and religion of their country of origin is considered in the public school system of the resident country and (d) their preference for a marriage partner from the own cultural community.
**Hypothesis 6:** Immigrants, even those who qualify as biculturals according to their self-categorization or their behavioral adaptation, typically identify with one sociocultural group on the level of norms and values (either the culture of origin, or the culture of the country of residence).
**Supportive findings.** For our sample of 1^st^ to 2^nd^ generation immigrants, we expected the norm identification group to be mostly still the culture of origin. In support of hypothesis 6, the majority of the participants—even of those who were clear biculturals according to their self-categorization or behavioral adaptation—reported that their values are still those of their culture of origin, and that they fairly often experience cultural value conflicts.
**Hypothesis 7.** Immigrants, like all sociocultural groups, believe that members of their own and other sociocultural groups have a culture-transmission motive.
**Supportive findings**. Supporting hypothesis 7, most participants believed that everybody wants to see his or her culture of origin live on in the next generation.
**Hypothesis 8.** Immigrants, like all sociocultural groups, believe that they and other sociocultural groups have the moral right to transmit their culture.
**Supportive findings**. In the present study, hypothesis 8 was tested for migrants as reference groups. In support of the hypothesis, most participants believed that cultural transmission is a moral right of immigrants.

### A Theory of Cultural Transmission in Minorities

The focus of the theory of cultural transmission in minorities [14,16] are the interactions between sociocultural majorities and minorities in the area of education: the educational activities of minorities undertaken for the purpose of maintaining their culture, and the educational policies of the majority towards them. (In accordance with a common definition of ethnic minorities in contemporary social science (e.g. [24,25]), cultural minorities are conceptualized as low-power subgroups of a society that have or claim a cultural (ethnic, linguistic, religious) identity.) To explain these interactions, we make the simplifying methodological assumption that group interactions can be modeled as analogous to interactions between individuals. Accordingly, we conceptualize the minority and the majority as two social actors who are equipped with particular beliefs, desires (goals), and resources (power, skills, material resources, etc.) and who attempt, by and large in a rational fashion, to achieve their goals in the area of education in a given historical situation. As in the case of the interaction between individuals, the actions of the minority and the majority and their success or failure are assumed to be determined by three groups of factors (see e.g. [26–29]): (1) motivational factors, i.e. the motives, desires, or goals of the minority and majority; (2) epistemic factors, in particular the beliefs of the minority and majority about their ability to realize their goals by particular actions; and (3) the objective situational constraints that apply to both actors (e.g., knowledge, financial resources, laws).

In the theory of cultural transmission in minorities, this general action-theoretical model of group interaction is elaborated by supplementing it with additional assumptions concerning, among others, the typical motives and strategies of minorities and majorities in cultural transmission situations. The most important of these assumption is that members of sociocultural groups typically have, in addition to their other motives, a *culture-transmission motive*: The (explicit or implicit) desire to preserve the culture of origin and transmit it to the next generation. This motive is carried by migrants to the country of immigration and (due to its relative stability; see below) is hypothesized to be still active in the majority of immigrants of (at least) the 1^st^ to 2^nd^ generation (Hypothesis 1, see Table 1). Therefore, explanations of migration-related phenomena that fail to consider the culture-transmission motive are believed to be incomplete.

Inspired by a theory of cultural evolution proposed by D. S. Wilson [30,31] and in similar form by Richerson and Boyd [32], Mchitarjan and Reisenzein [14] assume that the culture-transmission motive (1) has originally emerged in the course of cultural evolution as a mechanism that aids cultural transmission and (2) has been shaped by the cultural evolution process to focus on those elements of culture in a broad sense (i.e., the totality of socially transmitted information [32]) that are particularly important for the functioning of cultural groups (Hypothesis 2; Table 1). These core cultural elements are assumed to comprise, on the one hand, the values and norms of the group and the ideology that supports them, such as beliefs about a common origin and a shared destiny; and on the other hand, group characteristics that are reliable outward signs of a person’s cultural belonging and thereby allow group members—the carriers of the same cultural values and norms—to recognize each other. These characteristics include, importantly, the group’s language or sociolect (for evidence, see e.g. [33]). In addition, language is also of fundamental importance for cultural transmission because it is the central channel for the transmission of cultural information. Furthermore, the core elements of the transmitted culture are assumed to comprise the culture-transmission motive itself.

More specifically, the theory of cultural transmission in minorities assumes that the culture-transmission motive is transmitted together with specific cultural values and norms during socialization. This is achieved by social learning practices, largely completed before adolescence, that firmly anchor the culture-transmission motive in more basic motives typically fulfilled by groups: Individuals are explicitly or implicitly taught, as part of the socialization process, that adherence to the cultural system of the group provides them with a sense of identity and belonging, cognitive guidance, security, and the appreciation and support of the group members (e.g. [34,35]). If this assumption is correct, the strength of the culture-transmission motive should be predictable from the perceived instrumentality (see e.g. [36]) of the own cultural group for the fulfillment of these motives (Hypothesis 3, Table 1).

Because the learning experiences that establish the culture-transmission motive in the members of cultural groups are hard to undo, the desire to maintain and transmit the culture of origin is hypothesized to remain largely stable across the individual’s lifetime, similar to core values (e.g. [37]). This is also predicted to be the case for migrants, particularly those that emigrated as adolescents or adults (see also [38]). (Hypothesis 4a, Table 1). Furthermore, because the theory assumes that the culture-transmission motive belongs to the core of transmitted culture, it predicts that the motive will be transmitted to the next generation with similar efficiency as are core cultural norms and values (Hypothesis 4b, Table 1).

According to the theory of cultural transmission in minorities, the culture-transmission motive (like other motives) is not constantly present as a conscious desire in the minds of the members of sociocultural groups. Rather, it is conceptualized as a latent concern [39]) of which group members become aware only under special circumstances, especially if they perceive or suspect a threat to the transmission of their culture. This is assumed to occur regularly when members of a sociocultural group come into the sphere of influence of a different, more powerful group, as is typically the case in migration situations. Hence, in a cultural majority environment, the culture-transmission motive of the minority is activated. In this activated form, it then motivates actions (specifically actions of a pedagogical nature) aimed at countering the perceived threat to cultural transmission, such as “cultural” education in the family or the founding of own schools (Hypothesis 5a; Table 1). In addition, the activated culture-transmission motive generates specific desires that aid cultural transmission in the migration situation, such as the desire for support of one’s cultural transmission efforts by the majority [14] and the preference for a marriage partner from the own cultural community [15] (Hypothesis 5b; Table 1).

The assumption of the theory that the culture-transmission motive focuses on specific cultural elements (values and norms, language etc.) implies that immigrants can adopt many aspects of the majority culture (e.g., technological innovations; use of the majority language in everyday interactions), and as a consequence can integrate well into the majority society, particularly its job market and educational sector, without abandoning their primary identification with the culture of origin. In contrast, the adoption of norms and values of the majority culture is predicted to be more difficult. Specifically, conflicting norms and values usually cannot be integrated into one’s own normative system, but require a shift or “conversion” from one cultural value system to the other [14]. This leads to the prediction that “bicultural identities” (e.g. [40,41]) at the level of norms and values are the exception rather than the rule, even among immigrants who categorize themselves as biculturals (i.e. regard themselves as belonging to both cultures [42]) and are behaviorally well-adapted to both cultures (“behavioral biculturals” [43]). Rather, on the level of norms and values, immigrants are predicted to identify primarily with one sociocultural group. The theory of cultural transmission in minorities thus suggests a refinement of existing models of biculturalism [43,44]: While bicultural identities are predicted to be more or less frequent on the level of self-categorization and the level of behavior, on the level of norms and values, identification with one sociocultural group is expected to be typical. For 1^st^-2^nd^ generation immigrants, given their still strong motive to maintain the culture of origin, this norm identification group is predicted to be still mostly the culture of origin (Hypothesis 6; Table 1).

If it is true that sociocultural groups have a culture-transmission motive that has important motivational effects, this fact should not have gone unnoticed in the history of mankind. Based on this consideration, we propose that the assumption of the existence of a culture-transmission motive is part of the folk psychology of sociocultural groups: Social actors believe that the members of their own and of other sociocultural groups value their cultures and desire to maintain them (Hypothesis 7; Table 1). Furthermore, given that it is difficult to legitimately deny other groups that which one desires for one’s own, we predict that the mutual knowledge of groups about their desire for cultural transmission has resulted in an intuitive moral approval of cultural transmission efforts [17]. That is, social actors believe that they and other sociocultural groups have the moral right to transmit their culture (Hypothesis 8; Table 1).

### Aims of the Present Study

The aim of the study reported in this article was to test the eight hypotheses described in the preceding section in a survey of a relatively large and diverse sample of 1^st^ to 2^nd^ generation immigrants. The hypotheses are again summarized in Table 1.

## Materials and Methods

### Ethics Statement

The survey was conducted in accordance with the World Medical Association’s Declaration of Helsinki and the Ethical Guidelines of the German Association of Psychologists (DGPs) [45]. Formal ethics approvals for surveys of the type reported in this paper are not required by these guidelines. At the time of the study (2013) it was also not customary at the University of Greifswald and most other German University to seek ethics approval for anonymous surveys. The study was judged by the authors to not cause any harm or distress to the participants. Based on this assumption, which according to German laws is at the discretion of the authors and for which they hence assume full responsibility, and in line with the above-mentioned guidelines, a formal ethics approval was not required and hence not requested. The study used an anonymous questionnaire, hence neither the name nor the email address was obtained from the participants; the IP addresses were collected by the questionnaire software, but were deleted prior to analysis. Participation was voluntary. Participants were informed that the questions concerned the role played by the family and culture of origin in their lifes, and about the anonymity of the data, and they could withdraw from the study at any time. Participation was thus based on implicit informed consent; non-consenting individuals did not produce data or returned an incomplete questionnaire. Participants were given the opportunity to comment on the questionnaire at the end of the study; 25% made use of this opportunity, and nearly all expressed a favorable evaluation of the survey.

### Questionnaire

The hypotheses summarized in Table 1 were tested using a modified version of a questionnaire developed by Mchitarjan and Reisenzein [5]. Items of the original questionnaire not relevant to the hypotheses were eliminated, but several new items were added to test hypotheses 7 and 8 and to improve the tests of some of the remaining hypotheses. The original answer format of the items was changed to a common 8-point (0–7) rating scale whenever possible; however, the labels of the scale endpoints were specific to each item or item group [46]. The items are described in the Results section and in S1 Text. The participants were first asked to provide biographical information; the remaining items were presented in a partially randomized order aimed at encouraging independent responses to similar questions. The questionnaire was implemented as a web survey using the online questionnaire generator tool *SoSci Survey* [47]. Data collection was conducted on the soscisurvey platform (http://soscisurvey.de). Completing the questionnaire took 10–15 minutes.

### Recruitment of Participants

Participants were recruited at microworkers.com (www.microworkers.com), an online microjob platform similar to Amazon Mechanical Turk [48,49]. Microworkers was preferred as a recruitment site for the present study because its users represent a broader international audience [48]. Microworkers offers registered users the opportunity to complete online tasks including surveys in return for small payments, as well as to offer such jobs themselves. Payment depends on the task and the user’s country of residence (to take income differences between countries into account), but typically ranges from $0.10 to $2. In our study, the participants were offered from $.60 to $1.50 in different countries, as proposed by the microworkers payment calculation system.

The task description stated that we are looking for people with a migrant background to complete a survey about the role played by the family and culture of origin in their lifes (see S1 Text). “Having a migrant background” was operationally defined as “you were not born in the country where you now live, *or* your mother, father or both were not born in the country where you were brought up and where you now live”. Hence, our study focused on 1^st^-2^nd^ generation immigrants [38]. Those who wished to participate followed the link to the questionnaire at soscisurvey.de, completed the questions, and then returned to Microworkers to verify their participation.

With an estimated 450.000 registered users at the time of data collection (2013), we could expect Microworkers to comprise a sufficient number of persons with a migrant background to obtain a sample of the desired size. Microworkers allows to place jobs (called “campaigns”) in individual countries, as well as in clusters of countries grouped (at the time of the study) into five world regions: “Europe East”, “Europe West”, “USA/Western” (comprising the US, Canada, the UK, and Australia), “Asia and Africa”, and “Pacific”. Our goal was to obtain at least 500 participants from a diverse set of countries and cultures. To achieve this aim, we started five separate campaigns, each with a target sample size of 250, in the five world regions. Within a few weeks, the desired sample size was reached in all regions with the exception of Western Europe and the Pacific region. Because the target sample size in these regions was not reached even after 2.5 months (at that time, 200 participants from Western Europe and 39 from the Pacific region had been recruited), we decided to stop data collection at this point.

### Data Screening

In addition to fulfilling the operational definition of “people with a migrant background”, participants included into the data analyses had to have largely complete data and sufficient proficiency in English. We first eliminated participants who had omitted one or more pages of the questionnaire, had more than 7% missing response, or had apparently “clicked through” the questionnaire (to identify these, we searched for subjects with very low rating variance and then visually inspected a line plot of their ratings). This left 1083 participants with useable data. Next, we discarded participants with scores below the midpoint of the mean of two self-ratings, made by the participants at the end of the questionnaire, of their general knowlegde of English (0 = “minimal” to 7 = “excellent”) and their understanding of the questions (0 = “barely” to 7 = “completely”). This reduced the sample size to 998. Finally, we excluded participants who did not fulfill the operational definition of people with migrant background, as determined from their responses to the biographical questions asking for country of birth, upbringing, and residence, and the countries of birth of their parents. This led to the exclusion of another 154 participants, resulting in a final sample size of 844. The self-rated English proficiency of the included participants was very high (*M* = 5.76, *SD* = 1.22), as was their self-rated question understanding (*M* = 6.21, *SD* = 1.11), suggesting that their English knowledge was adequate for the task. This conclusion is supported by the high education level of most participants, as well as the high obtained correlations between multiple items measuring the same constructs (see Results).

### Data Analysis

The data were analyzed using R [50]. Prior to the analyses, remaining missing values on variables used in the analyses were estimated using *K*-Nearest-Neighbor imputation (*k*NN, e.g. [51]), as implemented in the VIM package of R [52]. In *k*NN imputation, a missing value of a case *i* on a variable *X* is replaced by—depending on the scale level of *X*—the mean, median or mode of the *X*-values of the *k* cases most similar to *i* on the nonmissing variables (we used *k* = 5). *k*NN imputation has been found to perform well compared to more complex imputation procedures [51].

## Results

### Sample Description

**Fig 1:**
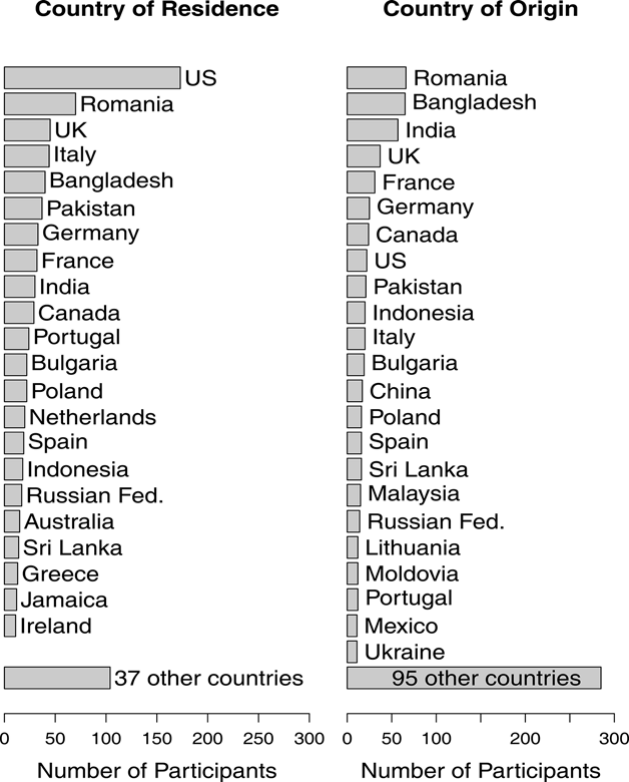
Number of participants living in, and stemming from, different countries.

620 of the 844 participants were men and 224 women, with a mean age of 27.6 years (*SD* = 8.4). The immigrants were living in 59 different countries, 22 of which contributed more than 10 participants (see Fig 1). Duration of stay in the country of residence was on average *M* = 14.8 years (*SD* = 10.5).

The participants’ or their parents’ country of origin was inferred by the authors from their responses to the demographic items (country of birth, upbringing, and residence; parents’ countries of birth) and their responses to a free cultural self-description question (e.g., “Vietnamese-American”). If only one parent stemmed from the country of residence, the country of origin was that of the foreign-born parent; if the parents had different foreign nationalities, that of the mother was used [38]. According to this information, the participants or their parents stemmed from 118 different countries, 23 of which contributed more than 10 participants (Fig 1).

31.5% of the participants came to the country of residence as adults (18 or older) and thus qualify as 1^st^ generation migrants [38], 18.5% were born in the country of residence (2^nd^ generation), and the remaining 50% were born abroad but were brought up in the country of residence. According to a more fine-grained classification of the latter group proposed by Rumbaut [38], which is based on the immigrants’ age at the time of entry to the resident country, 10% belonged to generation 1.25 (age at time of entry 14–17 years), 19% belonged to generation 1.5 (7–13 years), and 21% to generation 1.75 (6 years or younger).

Concerning religion, 40% of the participants classified themselves as Christians, 21% as Muslims, 7% as Buddhists, 5% as Hindus, 1% as Jewish, 7% had another religion, 11% called themselves atheists and 9% agnostics. Regarding education level, 44% of the participants had a university degree, 41% had a certificate that qualified for study at a university, 12% had a lower certificate and 3% had no (or not yet a) school certificate.

### Measurement of the Culture-Transmission Motive

The participants’ culture-transmission motive was measured using a slightly modified version of the multi-method motive measure developed by Mchitarjan and Reisenzein [5]. This measurement instrument is based on the assumption that motives (desires, preferences) can be assessed both *directly*, by asking people about the strengh or importance of the desire of interest; and *indirectly*, by inferring motive strength from positive or negative emotions experienced if the desire is fulfilled or frustrated, or from actions in which the motive manifests itself particularly clearly (see also [53,54]). For the present study, a few redundant items were removed from the original version of the culture-transmission scales and several new items were added to improve the measurement of the desire to transmit values and norms.

#### Direct measurement of the culture-transmission motive

Three items were used for the direct measurement of the culture-transmission motive: “How important is it for you to keep the culture of origin of your family and to pass it on to your children?”; “How important to you are the values and norms of your family’s culture of origin—its ideas about the right way to live, its beliefs about what is proper and what not?”; and “Do you wish that your family’s culture of origin is kept alive in the generation of your (present or future) children?”. All questions were answered on 0–7 point rating scales, with endpoints labeled 0 = “not important at all”, to 7 = “very important” for the first two items, and 0 = “I do not care at all” to 7 = “I wish this very much” for the third item. A principal axis factor analysis [55] of the items, using the R package *psych* [56] was consistent with a one-factor solution, revealing high loadings (.75-.85) for all items. To evaluate the replicability of the factor solution, the sample was split in two random subsets, the factor analysis was repeated for each subset, and the factor loadings were compared (see [57]). Nearly identical loadings were obtained. (Parallel replicability analyses were performed for the factor, correlation and regression analyses reported later. In all cases, very similar results were obtained for the two split-half samples). The internal consistency (Cronbach’s α) of the scale CTM-Desire, formed as the mean of the three items, was .84.

#### Motive-diagnostic emotions

Two groups of items were used to infer the culture-transmission motive from diagnostic emotions. First, the participants rated how much they would regret the loss of central elements of the culture of origin (language, values and norms [2 items], and religion of the family) in the generation of their children, as well as the complete disappearance of the culture in the future, on scales ranging from 0 = “would not mind” to 7 = “would feel very sorry”. Second, the participants reported the degree of happiness elicited by positive media reports about their family’s country or culture of origin, and the degree of anger elicited by negative media reports (0 = “I don’t mind at all” to 7 = “this makes me very happy [angry]”). To evaluate the homogeneity of the seven emotion items, they were subjected to a principal axis factor analysis with correlated factors (oblique rotation; we used the oblimin method), with the number of factors determined using the scree plot (which seems advantageous in large samples [57]). The scree plot suggested two factors that together explained 49% of the item variance. All items with the exception of the two measuring emotional reactions to media reports loaded most strongly on the first factor; the latter items loaded on the second factor. However, because the two factors were strongly intercorrelated (*r* = .73), we deemed it allowable to combine all items into a single scale (CTM-emotion). The internal consistency of CTM-emotion was Cronbach’s α = .83.

#### Motive-diagnostic actions

Three items were used to infer the culture-transmission motive from actions (or action tendencies) considered to be diagnostic for this motive (a) the participants’ preference for a marriage partner from the culture of origin (see e.g. [22,58]); (b) the reported preference of their parents concerning their marriage partner (considered to be an indicator of their willingness to act in agreement with their family’s wishes; see [36]); and (c) the felt obligation to help a member of the culture of origin who is in trouble. (A fourth item used by Mchitarjan and Reisenzein [5] was excluded because of low correlations). A factor analysis of the items was consistent with a one-factor solution; the resulting three-item scale (CTM-Action) had a consistency of α = .73.

Similar to Mchitarjan and Reisenzein [5], the direct motive measure (CTM-desire) was strongly correlated to CTM-emotion (*r* = .78), and both scales correlated .54 to CTM-action. These correlations are probably as high as one can expect them to be, considering that the three scales implemented different motive measurement methods, each which its method-specific variance, and that the items of the three scales partly addressed different facets of culture. Furthermore, as in Mchitarjan and Reisenzein [5], the correlations of the direct motive measure to other variables were nearly identical to those of a scale formed by combining all items (CTM-total, α = .90; correlation to CTM-Desire = .88). For this reason, we used, as in Mchitarjan and Reisenzein [5], the direct motive measure (i.e. the CTM-desire scale) to test hypotheses about the relations of the culture-transmission motive to other variables.

### Existence and Content of the Culture-Transmission Motive (Hypotheses 1 and 2)

**Fig 2:**
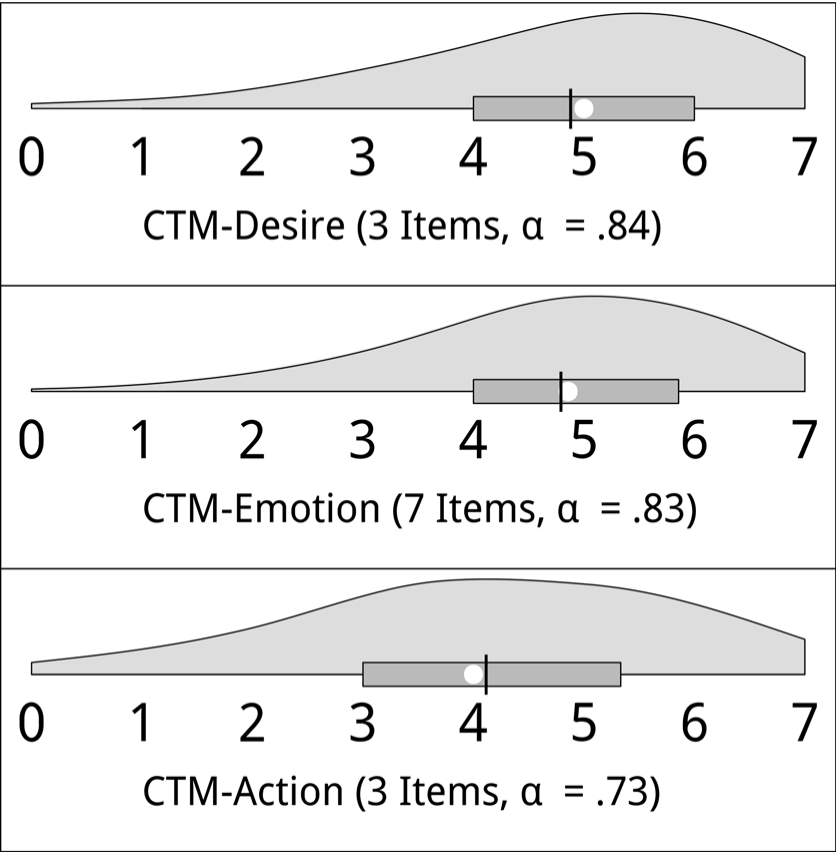
Distribution of the scale values (violin plots) of the desire, emotion and action subscales of the culture-transmission motive (CTM) measure. Higher values reflect a stronger culture-transmission motive. The white circle represents the median and the black bar the mean; the box ranges from the first to the third quartile. Answer scales were item-specific (see text).

Hypothesis 1, in specified form, asserts that the majority of 1^st^ to 2^nd^ generation immigrants still have the desire to maintain and transmit their culture of origin (Table 1). This hypothesis received strong support: The means and medians of all items used to measure the culture-transmission motive were far beyond the natural zero points of the respective response scales (indicating complete absence of the motive), with means ranging from 3.58 to 5.22 and medians from 4 to 6. Parallel results were obtained for the three scales formed from the items: CTM-desire (*M* = 4.88; *SD* = 1.56; *median* = 5), CTM-emotion (*M* = 4.79; *SD* = 1.38; *median* = 4.86) and CTM-action (*M* = 4.11; *SD* = 1.69; *median* = 4). More detail is given in Fig 2, which shows violin plots [59] of the distribution of the scores on the three motive scales. A violin plot combines a boxplot [60] with a representation of the estimated density function of the variable (essentially a smoothed histogram), thus enriching the boxplot with more detailed information about the shape of the distribution. As can be seen, the scale values of nearly all participants were beyond the zero point of the scales and the clear majority (82%, 83% and 66%, respectively) were beyond the midpoint (3.5).

Hypothesis 2 asserts that the culture-transmission motive focuses on the values and norms of the culture of origin, as well as on reliable outward signs of cultural identity, in particular the language. This hypothesis implies that items measuring wishes to transmit specific core elements of culture (language, values and norms) and items measuring the desire to transmit the culture in general, are strongly intercorrelated—as high as permitted by random measurement error and the fact that partly different measurement methods were used. In other words, these items should represent a single factor. To test this prediction, the specific and general motive items included in our measurement instrument (the three items making up the CTM-Desire scale and the first five items of the CTM-Emotion scale) were subjected to a principal axis factor analysis with oblimin rotation. Although the scree plot suggested two factors, the factors were strongly intercorrelated (*r* = .71) and a single factor explained nearly as much of the item variance (50% vs. 56%). Furthermore, all items had from acceptable (.51) to high (.83) loadings on this factor, which is thus clearly the general culture-transmission factor predicted by the theory.

### Anchoring of the Culture-Transmission Motive in Basic Motives Fulfilled by Groups (Hypothesis 3)

The theory of cultural transmission in minorities assumes that the culture-transmission motive is acquired during socialization by learning experiences that anchor the attachment to one’s culture and its representatives (the cultural group) in more basic motives fulfilled by groups. That is, the culture-transmission motive is derived from these more basic motives. To test this hypothesis (hypothesis 3), we asked the participants to which degree they ascribed commonly proposed social functions of groups (e.g. [34,35]) to their culture of origin, using seven items: my culture of origin reminds me of my roots, gives me orientation in my life, gives me the feeling of being able to rely on group members and their support, gives me a sense of protection, gives me a feeling of security, gives me stability, and gives me the feeling of being appreciated by others. Answers were given on a 0 = “do not agree at all” to 7 = “agree completely” scale. Subsequently, we predicted CTM-Desire from the social function ratings, using multiple regression. If the immigrants’ wish to transmit the culture of origin is based on their (implicit) belief that the culture fulfills these motives, then its strength should be predictable from the perceived instrumentalities (e.g. [36]). That is, the more immigrants believe that the culture of origin fulfills their desires for identity, orientation, social support etc., the more should they wish to maintain and transmit this culture.

As expected, all social functions of groups were ascribed by the participants to the culture of origin to some degree, with means ranging from 4.14 (gives me a sense of protection) to 5.20 (reminds me of my roots). The ratings were from moderately (.44) to strongly (.80) intercorrelated, suggesting that the culture of origin tends to play all functions simultaneously. Most important, the strength of the culture-transmission motive could be well predicted from the social function ratings, multiple *R* = .72, *p* < .001. Five of the seven social function ratings were significant predictors in the regression, the most important being “my culture of origin reminds me of my roots” (β = .41), followed by “gives me a sense of protection” (β = .26), “gives me orientation in my life” (β = .25), “gives me stability” (β = .23), and “gives me the feeling of being appreciated by others” (β = .14).

### Stability of the Culture-Transmission Motive (Hypothesis 4)

Hypothesis 4 asserts that the culture-transmission motive is (4a) intragenerationally highly stable (because it is firmly “implanted” during socialization); and (4b) has an intergenerational (parent-child) stability comparable to that of core transmitted norms and values (because it is part of the core transmitted culture).

To estimate the *intragenerational stability* of the culture-transmission motive in our cross-sectional sample, we correlated the participants’ motive strength with the length of their stay in the country of residence. This correlation estimates the stability of the motive in terms of its resistance to influences of the culture of the country of residence: If the immigrants’ desire to transmit their culture of origin is stable (resistant to change), it should be only little reduced with increasing duration of exposure to the majority culture, and hence its correlation with length of stay should be low. In agreement with this prediction, the correlation was *r* = -.10, *p* = .0057.

To estimate the *intergenerational stability* of the culture-transmission motive, we followed the typical procedure of research on intergenerational value transmission (e.g. [61,62]): We correlated the participants’ culture-transmission motive with that of their parents. Because a direct measure of the parents’ culture-transmission motive was not available, its strength was inferred from the participants’ answers to three questions: “How important is it or was it for your parents to keep their culture of origin and pass it on to their children?” (*M* = 5.04, *SD* = 1.81), “…that you should learn the language of your family’s culture of origin?” (*M* = 5.06, *SD* = 1.92), and “How regularly were the traditions, festivities, rituals, and customs of your family’s culture of origin practiced in your parent’s house?” (*M* = 4.63, *SD* = 1.79). The resulting three-item scale (Cronbach’s α = .77) correlated .67 (*p* < .001) with CTM-desire, suggesting substantial intergenerational stability of the culture-transmission motive. In fact, parent-child correlations of norms and values are typically lower (e.g. [61–64]; the exception are religious values, e.g. [65]). Keep in mind, however, that the parents’ culture-transmission motive was only indirectly assessed (i.e., as it was perceived by the participants).

### Coping with Threats to Cultural Transmission (Hypothesis 5)

Hypothesis 5 asserts that the culture-transmission motive is activated in migration situations and then (5a) motivates actions (specifically of a pedagogical nature) and (5b) generates desires that help immigrants to cope with the increased difficulty of cultural transmission under the conditions of migration.

To test hypothesis 5a, the participants were asked which countermeasures they would take in case of a threat to their cultural transmission posed by two main challenges to cultural transmission in migration situations: detracting influences of the local culture (“you notice that your child begins to drift away from the family’s culture of origin”), and the unavailability of important cultural transmission channels in the country of residence (specifically, there is no possibility for the child to learn the language of origin in the public school system). In the first scenario, they were additionally asked how they would react emotionally.

Regarding hypothesis 5b, we focused on two specific desires that the theory assumes will be generated by the culture-transmission motive in migration situations [14,15]. The first is the wish of immigrants that their cultural transmission efforts are supported by the majority society. To assess this wish, the participants were asked to which degree their culture and country of origin should be considered in the “ideal school” of their country of residence. The second wish is the preference for a marriage partner from the culture of origin. This wish aids cultural transmission because transmission is more likely to succeed if both parents stem from the culture of origin [15].

#### Reactions to cultural estrangement of the child

**Fig 3:**
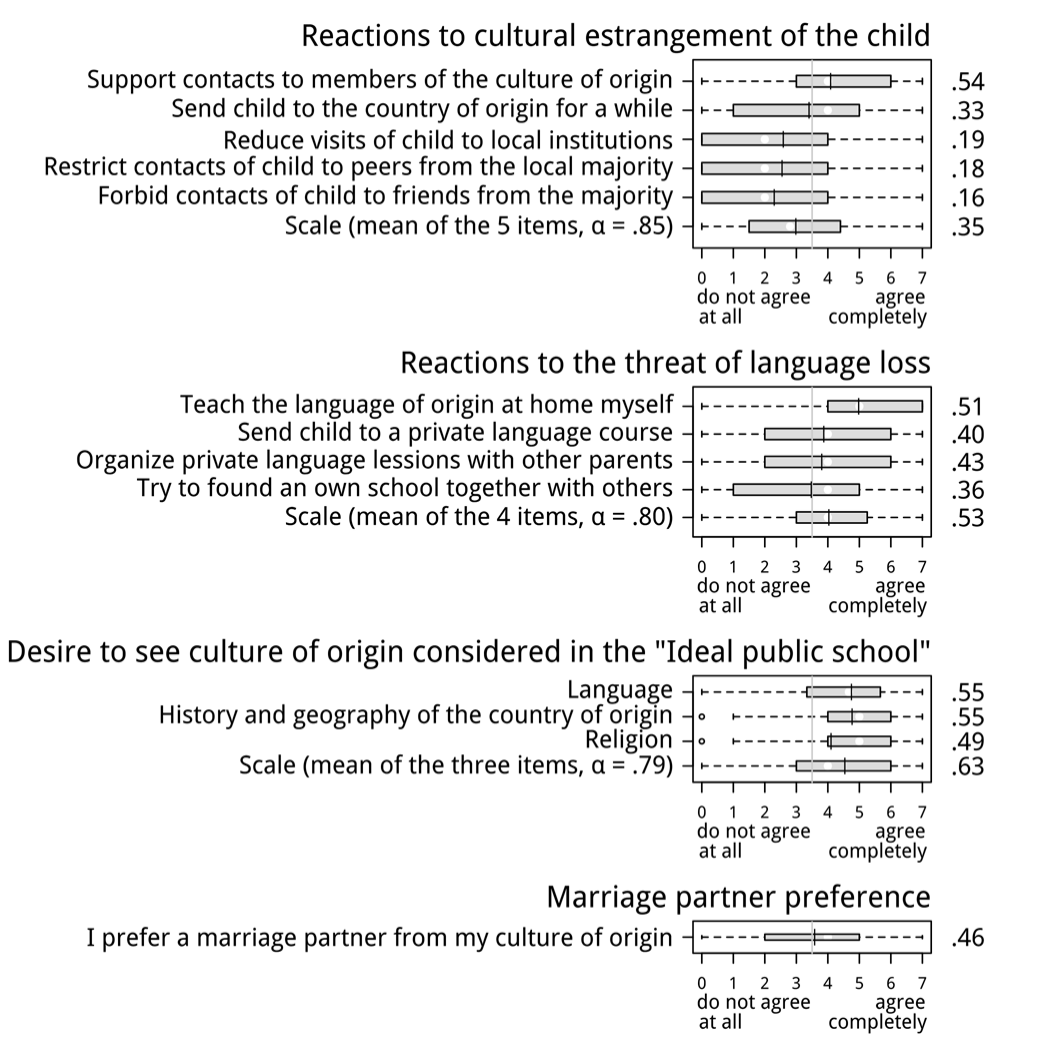
Effects of the culture-transmission motive on action tendencies and desires supportive of cultural transmission. Shown are boxplots of the distributions of the corresponding items. The median is represented by a white circle and the mean by a black bar; the box ranges from the first to the third quartile. The correlation of each item to the culture-transmission motive is listed next to the item on the right side of the figure. All correlations are significant at *p* <. 001.

Noticing that their child is drifting away from the culture of origin does not leave the participants emotionally unmoved; rather, most said that they would feel to some degree saddened (*M* = 4.29, *SD* = 1.98), disappointed (*M* = 4.09, *SD* = 2.09) and even alarmed (*M* = 3.99, *SD* = 2.16). Furthermore, as shown in Fig 3 (panel 1), most participants said that they would take one or more countermeasures against the perceived cultural estrangement of their child; the most preferred action was promoting contact of the child to members of the culture of origin. 80% of the participants agreed (scale value > 3.5) and 43% were certain or nearly certain (scale values 6 or 7) that they would take at least one of the countermeasures listed in Fig 3. Most important, both the emotional reactions and the action tendencies could be partly predicted from the participants’ culture-transmission motive, with correlations ranging from .50 (alarmed) to .64 (sad) for the emotions, and up to .54 for the action tendencies (Fig 3; all correlations are significant at *p* < .001).

#### Reactions to the threat of language loss

Most participants also said that they would take countermeasures against the danger of language loss due to the unavailability of language instruction in the public schools of the country of residence; the most preferred action was teaching the language of origin at home (Fig 3, panel 2). 90% of the participants agreed (scale value > 3.5) and 61% were certain or nearly certain (scores 6 or 7) that they would take at least one of listed actions. Again, the strength of the action tendencies could be partly predicted from the culture-transmission motive (*r*’s up to .54, all *p*’s < .001).

#### Desire for majority support of cultural transmission efforts

In addition to their readiness to engage in transmission-supportive activities themselves, most immigrants wished that their culture-transmission efforts are supported by the public school system of the resident country (Fig 3, panel 3). All items (wish for instruction in the language, history and geography, and religion of the country of origin) were fairly strongly endorsed and correlated significantly (*r*’s up to .63 for a scale formed as the mean of the three items) with the culture-transmission motive (all *p’*s < .001).

The findings concerning the language of origin were further supported by the answers given to an additional item that offered the participants a choice between six possible forms of school instruction distinguished by the increasing use of the language of origin. 92% wanted to see the language of origin considered in some form; 48% preferred instruction for their children in the language of origin, mostly with the language of the country of residence as an optional (18%) or compulsory (20%) subject; the remaining 52% preferred instruction in the language of the country of residence, mostly with the language of origin as a compulsory (20%) or an optional (24%) subject.

#### Marriage partner preference

An item measuring marriage partner preference was included in the action subscale of the CTM measurement instrument. 56% of the participants agreed (scale value > 3.5) that they preferred a marriage partner from the culture of origin (*M* = 3.58, *SD* = 2.21), and this preference correlated significantly (*r* = .46, *p* < .001) with the strength of their culture-transmission motive (CTM-Desire; see Fig 3, panel 4).

### Cultural Identity (Hypothesis 6)

**Fig 4:**
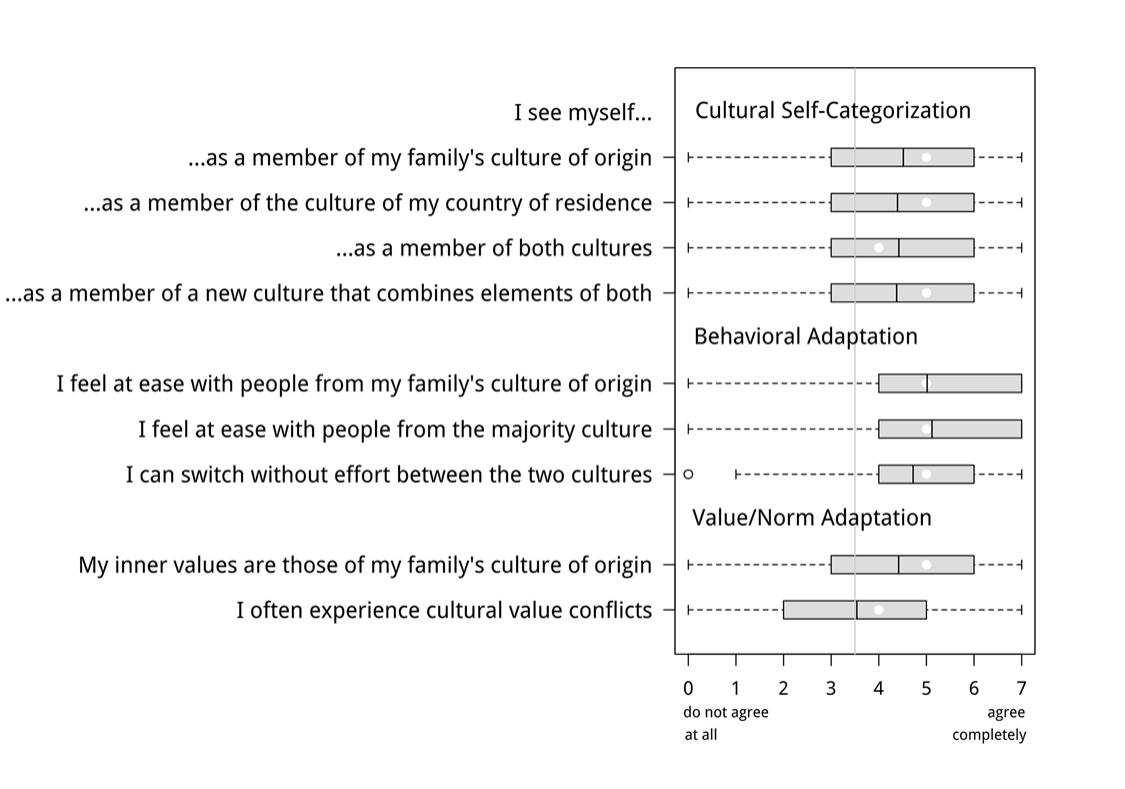
Self-ascribed cultural identity, adaptation on the behavioral level, and on the level of values and norms. Shown are boxplots of the distributions of the corresponding items. The median is represented by a white circle and the mean by a black bar; the box ranges from the first to the third quartile.

To test hypothesis 6 concerning the immigrants’ cultural identity, we asked them to rate their perceived belonging to the culture of origin and the culture of the host country, and we assessed their degree of adaptation on the level of behavior and the level of norms and values. The results are shown in Fig 4.

#### Self-ascribed cultural identity

To assess the participants’ self-ascribed cultural belonging, they were asked to indicate their agreement with the statements: “I see myself as a member of my family’s culture of origin” and “I see myself as a member of the culture of my country of residence”. Similar self-categorization measures have been frequently used in previous research on ethnic identity (e.g. [22,42]). As shown in Fig 4, a clear majority of the participants regard themselves as members of their culture of origin (71% > scale midpoint), and equally many see themselves as members of the majority culture (70% > scale midpoint), suggesting that the majority are self-ascribed biculturals. Supporting this conclusion, the majority also agreed to two additional items that explicitly asked them whether they regard themselves as belonging to both cultures (69% > scale midpoint), and as a member “of a new culture that combines elements of both cultures” (70% > scale midpoint; Fig 4).

#### Behavioral Adaptation

According to their self-reports, most participants also qualified as *behavioral biculturals*, defined as people who show “comfort and proficiency with both one’s heritage culture and the culture of the country or region in which one has settled” [43] (p. 26): Most said that they feel equally at ease with people from their family’s culture of origin (81% > scale midpoint) and with people from the host country (82% > scale midpoint), and that they can switch without effort between the two cultures (77% > scale midpoint; Fig 4).

#### Adaptation on the level of norms and values

The finding that most participants are bicultural in terms of their self-categorization and behavioral adaptation does not necessarily mean that they are also bicultural at a “deep” level, that is, have merged values and norms (e.g. [40]). Hypothesis 6 asserts that this is typically *not* the case; rather, on the level of norms and values, immigrants are predicted to identify primarily with one cultural group. For our sample of 1^st^ to 2^nd^ generation immigrants, given their still strong motive to maintain the culture of origin, we expected this norm identification group to be still mostly the culture of origin. Supporting this prediction, 70% of the participants agreed (scale values > 3.5) to the statement “Even if I adapt outwardly (in my behavior) to the culture of my country of residence, my inner values are those of my family’s culture of origin” (Fig 4). Furthermore, the stronger the participants’ culture-transmission motive, the more they agreed to this item (*r* = .56, *p* < .001). In addition, the majority of the immigrants agreed that they “often experience a conflict between the values of their country of residence and those of the culture of origin” (52% > scale midpoint; Fig 4), and again this item correlated significantly (*r* =. 28, *p* <. 001) with their culture-transmission motive.

To provide a stronger test of hypothesis 6, we examined the responses to the value items of “clear self-ascribed biculturals” (participants with scale values > 5 on the item “I see myself as a member of both cultures”; 35% of the sample) and “clear behavioral biculturals” (participants with scale values > 5 on the item “I can switch without effort between the two cultures; 38% of the sample). Again supporting hypothesis 6, 78% and 74% of these participants, respectively, agreed that their values are still those of the culture of origin, and 60% (55%) agreed that they often experience cultural value conflicts. In contrast, such conflicts were reported by only 27% (42%) of corresponding subgroups of self-ascribed and behavioral “monoculturals” (participants with scale values < 3 on the respective items). Both percentage differences are significant (Chi-square test for proportions, *p*’s < .001 and .05, respectively).

### The Folk Psychology and Folk Morality of Cultural Transmission (Hypotheses 7 and 8)

**Fig 5:**
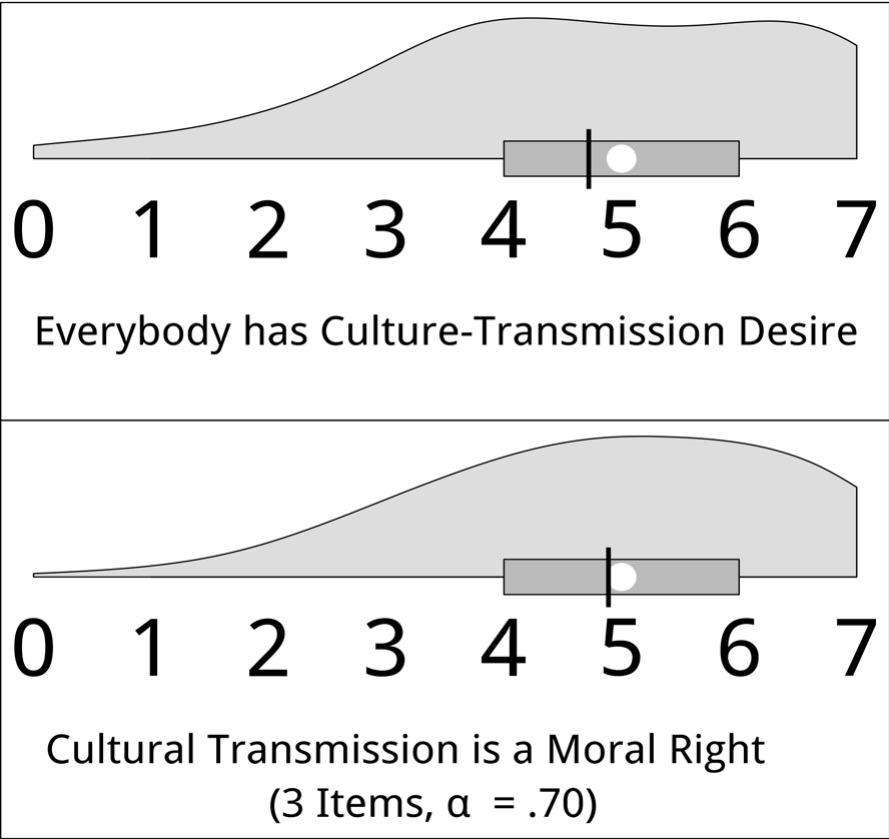
Distribution of the belief in the existence of the culture-transmission motive in others, and the perceived right of migrants to maintain their culture. Answers were given on scales ranging from 0 = “do not agree at all” to 7 = “agree completely”. The white circle represents the median and the black bar the mean; the box ranges from the first to the third quartile.

Hypothesis 7 asserts that the assumption that sociocultural groups have a culture-transmission motive is part of the folk psychology of groups. Supporting the hypothesis, most participants agreed to the item “Everybody values his or her culture of origin and wants to see it live on in the generation of his or her children”: *M* = 4.72, *SD* = 1.76, *median* = 5, 77% > scale midpoint. The distribution of the answers is shown in the upper panel of Fig 5.

Hypothesis 8 asserts that sociocultural groups regard the maintenance of culture as a moral right of groups; that is, the right to cultural transmission is part of the folk moral system of cultural groups. In the present study, we tested this hypothesis for migrants as reference groups, by asking the participants to which degree they agreed to the items: “It is wrong if migrants are forced to give up their culture of origin and to adopt the culture of their country of residence”, “I accept if people living in a foreign cultural environment want to keep their culture of origin”, and “Migrants should adapt to some extent to the culture of their country of residence, but need not give up their culture of origin”. The three items were sufficiently intercorrelated to combine them into a scale (α = .70). Supporting hypothesis 8, most participants believed that migrants have the right to maintain their culture (*M* = 4.89; *SD* = 1.50, *median* = 5, 81% > scale midpoint). Fig 5 (lower panel) shows the distribution of the scale values.

We also hypothesized that the mutual knowledge of sociocultural groups about their own and other groups’ desire for cultural transmission provides the cognitive basis for its moral justification, as it is difficult to legitimately deny other groups that which one desires for one’s own [17]. In agreement with this hypothesis, the “right to cultural maintenance” scale correlated *r* = .55 (*p* <. 001) with the participants’ belief that everybody has a culture-transmission motive, and *r* = .45, *p* < .001 with the participants’ own culture-transmission motive (multiple *R* = .59).

### Differences Between Subgroups

The theory of cultural transmission in minorities assumes that all sociocultural groups have a culture-transmission motive, and that it functions the same way for all. In agreement with this assumption of universality, we tested our hypotheses for the pooled sample of immigrants. Possible differences between subgroups of immigrants stemming from, or living in, different countries/cultures in the levels the measured variables or their intercorrelations, including differences caused by possible measurement inequivalence (e.g. [66]), were thus ignored. Note, however, that a major source of measurement inequivalence in cross-cultural surveys, translation error [66], was controlled because all participants completed the same English-language questionnaire. Furthermore, given that the sample was not strongly dominated by participants from a particular country of origin or residence (see Fig 1), possible systematic differences between subgroups of immigrants should only have moderate effects on the results obtained for the complete sample. As a partial test of this assumption, we compared the means and intercorrelations of the variables involved in the tests of the hypotheses (the culture-transmission motive, social functions ascribed to groups, action tendencies in case of threats to cultural transmission etc.) in the 11 countries (as a proxy for cultures) of origin that contributed at least 20 participants (see Fig 1). The means were compared using one-factorial analyses of variance. Although these analyses revealed statistically significant (*p* < .05) between-country differences for about half of the dependent variables, the corresponding effect sizes (η^2^) were moderate, ranging from 5% to 16% explained variance. For example, the between-group variance in the culture-transmission motive (CTM-Desire) was η^2^ = .063, with means ranging from 4.23 (immigrants from France) to 5.51 (immigrants from Pakistan). To examine the homogeneity of the within-group correlations, those of main relevance to our hypotheses (e.g., between the culture-transmission motive and the readiness to teach organize instruction in the language of origin for the child) were computed separately for the subgroups, averaged, and the averaged correlations were then compared to those obtained for the complete sample. In all cases, these correlations were highly similar.

Parallel findings were obtained when the sample was split according to sex, age (split by quartiles), immigrant generation [38], education level, and religious affiliation. For example, with respect to the culture-transmission motive (CTM-desire), no significant between-group differences (*p* < .05) were obtained for age and education level, and the variance explained by sex, immigrant generation status and religious affiliation was comparatively small, ranging from η^2^ = .026 (sex, *p* < .001), to .07 (religious affiliation, *p* < .001).

Finally, with respect to cultural differences, we emphasize that a moderate degree of between-culture heterogeneity is not only compatible with the present theory, but is predicted by all universalistic theories that, like the present one, posit mechanisms partly created by learning (see the discussion in Reisenzein [67]). In fact, the assumption that cultures differ in the level of the explanatory variables posited in these theories is a main way of how they account for cross-cultural differences in the phenomena they seek to explain. For example, intercultural differences in the strength of the culture-transmission motive, caused by different socialization practices, can partly explain differences in the readiness to engage in actions supporting cultural transmission, and as a result, differences in the success of cultural transmission in different groups of migrants.

## Discussion

We tested eight hypotheses derived from the theory of cultural transmission in minorities and obtained support for all hypotheses. The main supportive findings are summarized below each hypothesis in Table 1.

Hypotheses 1–6 were already tested in the previous study by Mchitarjan and Reisenzein [5] with a small sample of adolescents and young adults with mostly Russian migration background in Germany. The results were quite similar to those obtained in the present study, with differences being mostly quantitative in nature. For example, the median strength of the culture-transmission motive in the previous study was one scale point higher (*median* = 6) than in the present study (*median* = 5), but the correlations among the items measuring the culture-transmission motive, as well as the correlations of the culture-transmission motive to other variables, were for the most part equally high or higher in the present study. At the same time, the present study overcomes a main limitation of the study by Mchitarjan and Reisenzein [5], the small and restricted (in terms of both the culture of origin and the culture of the country of residence) sample. To be sure, our sample is not a representative sample of the worldwide population of 1^st^-2^nd^ generation immigrants either (it would certainly be very difficult to obtain such a sample). However, the sample does include immigrants stemming from, and living in, a large number of countries and cultures. Furthermore, as reported in the section “Differences between subgroups”, although there were statistically significant differences in the level of the culture-transmission motive and other variables between subgroups of immigrants from different countries—as well as between 1^st^ and 2^nd^ generation immigrants, males and females, and participants with and without religious affiliation—these differences were moderate in size, and the correlations between variables in the different subgroups were highly similar. Finally, as mentioned, our results agree closely with those obtained by Mchitarjan and Reisenzein [5] for yet another sample of migrants. The apparent robustness of our findings across groups suggests that the main pattern of our results will also hold true in other groups and subgroups of immigrants. Nevertheless, because even small differences between groups can be theoretically or practically important, future research should examine such differences in more detail.

In addition to these empirical arguments for the generalizability of our findings, we would like to reiterate (see [5]) that (1) for tests of *correlational* hypotheses (e.g., hypotheses concerning the correlations of the culture-transmission to other variables), a representative sample is not needed [68,69]; and (2) for tests of hypotheses about the *levels* of a variable (e.g., the presence or strength of the culture-transmission motive), a nonrepresentative sample is problematic only if it the selection criteria correlate with this variable. In our case, this could be the case specifically if the participants self-selected according to their culture-transmission motive (i.e. were more likely to participate if they had a stronger culture-transmission motive). While this objection can be raised against the previous survey [5], given that its participants were volunteers recruited on ethnoportals for Russian and Turkish immigrants, it is much less plausible for the present study, because the main motivation of workers on microjob platforms is to earn money [70].

The empirical support obtained in the present study for the theory of cultural transmission in minorities adds weight to the explanations offered by this theory for several migration-related phenomena that pose difficulties to existing theoretical accounts, in particular the often observed resilience of cultural traits in immigrants (see e.g. [1,15]). Specifically, immigrant parents seem to be no less successful in transmitting cultural norms and values to their children than parents of nonmigrant families, and migrants and their descendants very often continue to identify strongly with the culture of origin of their families [15]. The theory of cultural transmission in minorities attributes these phenomena to the activation of the culture-transmission motive in the migration situation and the resulting, special efforts of migrants to safeguard the transmission of their culture under the difficult conditions of migration. Hence, from the perspective of the theory of cultural transmission in minorities, these phenomena are not anomalies but are to be expected, because they are predicted by the theory.

## Supporting Information

S1 Text A pdf file containing the instruction of the questionnaire, the items used in the analyses, and the answer scales. (PDF)
