# Supplementary material for: The Culture-Transmission Motive in Immigrants: A World-Wide Internet Survey
Source: PLoS One. 2015 Nov 3;10(11):e0141625. doi: 10.1371/journal.pone.0141625 (PMC4631500; doi:10.1371/journal.pone.0141625)
Supplement: S1 Text — (PDF) [file pone.0141625.s001.pdf]

## Questionnaire Instruction and Items used

*Note: The items are here ordered according to topics/constructs corresponding to the article. In the actual questionnaire, which was implemented as a web questionnaire, the items were partly randomized as explained in the article.*

### Instruction

Dear participants!

We are a team of educational scientists and psychologists at the University of Greifswald, Germany. With this survey, we want to collect information about the role played by the family and the culture of origin in the life of people with a migrant background.

To participate in this survey, you must have a migrant background; that is, you must fulfill one of the following conditions:

1. You were NOT born in the country where you now live; OR
2. Your mother, your father or both were not born in the country where you were brought up and where you now live.

In addition, you need to know good English to be able to understand and answer the questions.

Answering the survey should take no more than 15 minutes. The survey is anonymous: You will not be asked to give your name or your email, nor any other information that could identify you. All data are treated confidentially. They are of interest to us only as group data.

Please read each question carefully and answer as precisely as you can. There are no "right" or "wrong" answers – we are only interested in your personal opinion.

Thank you for your support!

Principal Investigators, and responsible for the content:
PD Dr. Irina Mchitarjan (Educational Science), irina.mchitarjan@uni-greifswald.de
Prof. Dr. Rainer Reisenzein (Psychology), rainer.reisenzein@uni-greifswald.de

## Questionnaire Items

### Demographic Information

[Sex] Are you male or female?

- male
- female

[Age] In which year were you born? Please enter the year! [Variable Age was computed from these data]

[DurationStay] Since when do you live in your present country of residence? [Enter the year] [Variable DurationStay was computed from these data]

[CBirth] In which country were you born? [Select country from drop-down list]

[CBroughtup] In which country did you grow up? [Select country from drop-down list]

[CResidence] In which country do you live today? [Select Country from drop-down list]

[CBirthMother] In which country was your mother born? [Select country from drop-down list]

[CBirthFather] In which country was you father born? [Select country from drop-down list]

**[Cselfdescription] (Free cultural self-description)**
How would you complete the following sentence: "In my innermost core, I feel as a ..."? For example, if you are living in England, but have a Pakistani migrant background, use one of the following 4 answers: "...as a Pakistani" - "...as a British Pakistani" - "...as a Pakistani Englishman" --- "...as an Englishman". Please substitute your place of residence for 'England' and your country of origin for 'Pakistan', and answer according to what describes you best:

"In my innermost core, I feel as a ..." [Enter description]

**(Knowledge of English**, asked at the end of the questionnaire **)**
[EnglishKnowledge] How would you judge your knowledge of English?
minimal 0 1 2 3 4 5 6 7 excellent

[QuestionUnderstanding] How well did you understand the questions of the survey?
barely 0 1 2 3 4 5 6 7 completely

[Religion] What is your religion, or your nonreligious world view?

- Agnostic
- Atheist
- Buddhist
- Christian
- Hindu
- Jewish
- Muslim
- Other

[Education] What is your level of education?

- I have no school certificate
- I have a school certificate that does NOT qualify for study at the university

- I have a school certificate that qualifies for study at the university
- I have a university degree (e.g., Bachelor, Master, Diploma, Doctorate)

**(Measurement of the Culture-transmission motive)**

(**CTM-Desire**)
[CTMD01] How important is it for you to keep the culture of origin of your family and to pass it on to your children?
Not important at all 0 1 2 3 4 5 6 7 very important

[CTMD02] How important to you are the values and norms of your family's culture of origin – its ideas about the right way to live, its beliefs about what is proper and what not?
Not important at all 0 1 2 3 4 5 6 7 very important

[CTMD03] Do you wish that your family's culture of origin is kept alive in the generation of your (present or future) children?
I do not care at all 0 1 2 3 4 5 6 7 I wish this very much

**(CTM-Emotion**)

[CTME01] Would you feel sorry if your (present or future) children would forget or lose the language of your culture of origin?
would not mind 0 1 2 3 4 5 6 7 would feel very sorry

[CTME02] Would you feel sorry if your (present or future) children would turn their back on the religion of your family?
would not mind 0 1 2 3 4 5 6 7 would feel very sorry

[CTME03] Would you feel sorry if your (present or future) children would turn their back on the values and norms that are important to you?
would not mind 0 1 2 3 4 5 6 7 would feel very sorry

[CTME04] Would you feel sorry if your (present or future) children would turn their back on the values and norms that you adopted from your parents?
would not mind 0 1 2 3 4 5 6 7 would feel very sorry

[CTME05] A hypothetical question: Would you feel sorry if – for whatever reason – the culture of origin of your family would not exist anymore 500 years from now?
would not mind 0 1 2 3 4 5 6 7 would feel very sorry

[CTME06] Does it make you angry if the media (TV, radio, newspapers, magazines) of your country of residence report something negative or critical about your family's country or culture of origin?
I don't mind at all 0 1 2 3 4 5 6 7 this makes me very angry

[CTME07] Does it make you happy if the media (TV, radio, newspapers, magazines) of your country of residence report something positive or praiseworthy about your family's country or culture of origin?
I don't mind at all 0 1 2 3 4 5 6 7 this makes me very happy

**(CTM-Action)**
To what extent do you agree with the following statements?

[CTMA01] As my husband/wife, I prefer a man/woman from my family's culture of origin.

do not agree at all 0 1 2 3 4 5 6 7 agree completely

[CTMA02] As my husband/wife, my parents prefer a man/woman from the family's culture of origin.
do not agree at all 0 1 2 3 4 5 6 7 agree completely

[CTMA03] If an acquaintance from my culture of origin is in trouble, I should help him or her.
do not agree at all 0 1 2 3 4 5 6 7 agree completely

**(Basic Motives Fulfilled by Adherence to the Culture of Origin)**
What role does your family's culture of origin play in your life? In what ways is this cultural community important for your?

[BMotives01] It gives me stability.
do not agree at all 0 1 2 3 4 5 6 7 agree completely

[BMotives02] It gives me a sense of protection.
do not agree at all 0 1 2 3 4 5 6 7 agree completely

[BMotives03] It gives me orientation in my life.
do not agree at all 0 1 2 3 4 5 6 7 agree completely

[BMotives04] It gives me a feeling of security.
do not agree at all 0 1 2 3 4 5 6 7 agree completely

[BMotives05] It gives me the feeling of being appreciated by others.
do not agree at all 0 1 2 3 4 5 6 7 agree completely

[BMotives06] It gives me the feeling of being able to rely on other group members and their support. do not agree at all 0 1 2 3 4 5 6 7 agree completely

[BMotives07] It reminds me of my roots.
do not agree at all 0 1 2 3 4 5 6 7 agree completely

**(Consciousness of Culture of Origin)**
[Awareness] How often, in your daily life, do you become conscious of your (partly) different cultural background?
I never think of it 0 1 2 3 4 5 6 7 I am constantly aware of it

**(Reactions to Cultural Estrangement of the Child)**
How would you react if you notice that your (present or future) child begins to drift away from your family's culture of origin (e.g., the child no longer adheres to the cultural prescriptions about the 'right' way to live, or forgets the language of the culture of origin)?

[EmoEstrangement01] I would be sad about this.
do not agree at all 0 1 2 3 4 5 6 7 agree completely

[EmoEstrangement02] I would be disappointed about this.
do not agree at all 0 1 2 3 4 5 6 7 agree completely

[EmoEstrangement03] I would be alarmed by this.
do not agree at all 0 1 2 3 4 5 6 7 agree completely

[CSEstrangement01] I would try to reduce visits of my child to the institutions (e.g., schools, kindergartens, youth clubs) of the local majority culture.

do not agree at all 0 1 2 3 4 5 6 7 agree completely

[CSEstrangement02] I would try to support contacts of my child to people from my culture of origin.
do not agree at all 0 1 2 3 4 5 6 7 agree completely

[CSEstrangement03] I would try to restrict contacts of my child to peers from the local majority culture.
do not agree at all 0 1 2 3 4 5 6 7 agree completely

[CSEstrangement04] I would forbid contacts of my child to friends from the majority culture.
do not agree at all 0 1 2 3 4 5 6 7 agree completely

[CSEstrangement05] I would send my child for while to the country of origin of my family.
do not agree at all 0 1 2 3 4 5 6 7 agree completely

**(Reactions to Threat of Language Loss)**

What would you do if there is no possibility for your (present or future) child to learn the language of your culture of origin at school in your country of residence?

[CSLanguagLoss01] I would try to teach my child the language at home myself.
do not agree at all 0 1 2 3 4 5 6 7 agree completely

[CSLanguageLoss02] I would send my child to a private language course.
do not agree at all 0 1 2 3 4 5 6 7 agree completely

[CSLanguagLoss03] I would try to organize a private language course for my child together with other parents.
do not agree at all 0 1 2 3 4 5 6 7 agree completely

[CSLanguageLoss04] I would try to found an own school together with other parents or people from my cultural community.

**(Preferred Form of School Instruction)**

[PrefSchoolInstruction] What kind of schooling would you most desire for your (present or future) children in your country of residence (regardless of whether it is currently possible)? [select one]

- Instruction is in the language of my family's culture of origin; the language of the country of residence is NOT taught as a second language.
- Instruction is in the language of my family's culture of origin; the language of the country of residence is taught as an optional course.
- Instruction is in the language of my family's culture of origin; the language of the country of residence is taught as an obligatory course.
- Instruction is in the language of the country of residence; the language of origin of my family is taught as an obligatory course.
- Instruction is in the language of the country of residence; the language of origin of my family is taught as an optional course.
- Instruction is in the language of my country of residence; the language of my family's culture of origin is NOT taught as a second language.

(**Consideration of the Culture of Origin in the Ideal School**)
What would the ideal school for your (present or future) child look like?

[IdealSchool01] In the ideal school, my child would have the opportunity to learn the language of my culture of origin.
do not agree at all 0 1 2 3 4 5 6 7 agree completely

[IdealSchool02] In the ideal school, my child would learn about the history and geography of my family's country of origin.
do not agree at all 0 1 2 3 4 5 6 7 agree completely

[IdealSchool03] In the ideal school, my child would receive instruction in the religion of my country of origin.
do not agree at all 0 1 2 3 4 5 6 7 agree completely

**(Cultural Identity)**
**(A. Self-categorization)**
How would you describe your cultural identity?
[SelfCat01] I see myself as a member of my family's culture of origin.
do not agree at all 0 1 2 3 4 5 6 7 agree completely

[SelfCat02] I see myself as a member of the culture of my country of residence.
do not agree at all 0 1 2 3 4 5 6 7 agree completely

[SelfCat03] I regard myself as being equally a member of both cultures.
do not agree at all 0 1 2 3 4 5 6 7 agree completely

[SelfCat04] I regard myself as a member of a new culture that combines elements of both cultures.
do not agree at all 0 1 2 3 4 5 6 7 agree completely

**(B. Behavioral Biculturality)**
To what extent do you agree with the following statements?

[BehBicu01] I feel comfortable with people from my family's culture of origin.
do not agree at all 0 1 2 3 4 5 6 7 agree completely

[BehBicu02] I feel comfortable with people from the culture of my current country of residence.
do not agree at all 0 1 2 3 4 5 6 7 agree completely

[BehBicu03] I can switch without effort between my culture of origin and the culture of my country of residence.
do not agree at all 0 1 2 3 4 5 6 7 agree completely

**(C. Biculturality on the level of norms and values: high values reflect low biculturality)**

[NormsStillCO] Even if I adapt outwardly (in my behavior) to the culture of my country of residence, my inner values are those of my family's culture of origin.
do not agree at all 0 1 2 3 4 5 6 7 agree completely

[ValueConflicts] I often experience a conflict between the values of my current country of residence and the values of my culture of origin.
do not agree at all 0 1 2 3 4 5 6 7 agree completely

**(Parents' Culture Transmission Motive)**

[ParentCTM01] How important is it or was it for your parents to keep their culture of origin and pass it on to their children?
not important at all 0 1 2 3 4 5 6 7 very important

[ParentCTM02] How regularly were the traditions, festivities, rituals, and customs of your family's culture of origin practiced in your parent's house?
never 0 1 2 3 4 5 6 7 always

[ParentCTM03] How important is it or was it for your parents that you should learn the language of your family's culture of origin?
not important at all 0 1 2 3 4 5 6 7 very important

**(Folk Psychology and Folk Morality of Cultural Transmission)**

To which degree do you agree with the following claims?

[BeliefCTM] Everybody values his or her culture of origin and wants to see it live on in the generation of his or her children.
do not agree at all 0 1 2 3 4 5 6 7 agree completely

[MoralCTM01] It is wrong if migrants are forced to give up their culture of origin and to adopt the culture of their country of residence.
do not agree at all 0 1 2 3 4 5 6 7 agree completely

[MoralCTM02] Migrants should adapt to some extent to the culture of their country of residence, but need not give up their culture of origin.
do not agree at all 0 1 2 3 4 5 6 7 agree completely

[MoralCTM03] I accept if people living in a foreign cultural environment want to keep their culture of origin.
do not agree at all 0 1 2 3 4 5 6 7 agree completely

**(Comments on the Survey)**
[CommentsOnSurvey] Do you want to make any comments regarding this survey? If so, please enter your comments here (if no, please proceed) [Enter comments]
